# Supplementary material for: Characterization of dengue patients in Vietnam: Clinical, virological, and IL-10 profiles during 2021– 2022 outbreaks
Source: PLoS Negl Trop Dis. 2025 Mar 28;19(3):e0012954. doi: 10.1371/journal.pntd.0012954 (PMC11977983; doi:10.1371/journal.pntd.0012954)
Supplement: Attachment — Submitted filename: IL10_Response to Reviewers.docx [file pntd.0012954.s001.docx]

**Reviewer #1**: The authors are studying the biostatistical relationships between clinical, viral and IL-10 profiles of two consecutive Dengue outbreaks in Vietnam between 2021 and 2022.

The **study design** is complicated and less clearly explained than it could be. One of the key points seems to be that DENV infection and its clinical profile may be influenced by the interaction between the genetic component of the host and the DENV serotype.

Sentence-level writing is satisfactory; the problem is at a higher level of organization where I found it difficult to understand the logical and immunological flow of the manuscript.

Two of the main potential strengths of this study are that:

a) The study is carried out during two consecutive Dengue outbreaks in Vietnam.

b) Reports of genotypic and allelic frequencies vary between populations, as observed among patients from Vietnam and Mexico, for example. But these strengths are not evident in the introduction as currently written.

Main concerns: Regarding the last 2 paragraphs of the introduction, in the penultimate one it is not clear if the bibliographic **citation (13)** corresponds to the described assertion and in the last one it is not clear what is the novelty that was shown in this study in comparison with previous studies. Please clarify.

**Response:** We thank the reviewer for his constructive suggestions. Considering his comments and suggestions, we have addressed all questions and responded accordingly and are highlighted in the revised manuscript.

- This cross-sectional study was conducted during two consecutive seasonal dengue outbreaks in Vietnam in 2021 and 2022. The study population comprised patients admitted to hospital with dengue, with varying degrees of clinical severity. The study aimed to investigate the association of IL-10 plasma level, *IL-10* genetic variants with dengue. The study also updated the epidemiology of circulating DENV serotypes in Vietnam.

- The manuscript was reorganized at the paragraph level in order to enhance readability. The citations were revised to ensure consistency with the assertions made.

- The frequencies of IL-10 genotypes and alleles exhibit considerable variation between populations respecting the geographical regions. However, the Vietnamese population has not been extensively studied with regard to *IL-10* variants (SNPs rs1800896, rs1800871, rs1800872), and thus the observed differences were included in the discussion. The data were obtained from Ensembl.org and referenced in the manuscript.

**Reviewer #2:** The objectives of the study are clearly stated. The authors reported the incidence of dengue in two outbreaks that occurred in 2021-2022 and also investigated the contribution of IL-10 levels and genotypes to Dengue susceptibility.

The study design was appropriate for the stated objectives

**Response**: Thank YOU.

The population was clearly described

**Response**: Thank YOU.

The authors did not show how they arrived at the sample size.

**Response**: Thank YOU. Sample sizes were calculated based on previous outbreaks and incidences over years based on published data and based on the allele frequencies observed for the IL-10 SNPS for Vietnamese population form 1000 genomes database. Sample size n = [DEFF*Np(1-p)]/ [(d2/Z21-α/2*(N-1)+p*(1-p)],with a power of 80% and a two-tailed α=0.05, β=0,8.

Correct statistical methods were employed

**Response**: Thank YOU.

Yes, all ethical and regulatory requirements were well stated

**Response**: Thank YOU.

**Response:** We thank the reviewer for his constructive suggestions. Taking into account his comments and suggestions, we have addressed all questions and responded accordingly

- This cross-sectional study was conducted during two consecutive seasonal dengue outbreaks in Vietnam in 2021 and 2022. The study population comprised patients admitted to hospital with dengue, with varying degrees of clinical severity.

**Reviewer #1**: In the materials and methods section, it is important to add a section describing the inheritance models used in this study.

**Response:** We thank the reviewer for his constructive suggestions. Taking into account his comments and suggestions, we have addressed all questions and responded accordingly. The description of applied inheritance models, comprising Dominant, Recessive, and Co-dominant, were incorporated in the revised manuscript, as suggested accordingly.

*‘Inheritance model analysis: The association of IL-10 genetic variants with dengue was analyzed using logistic regression adjusted for age and sex under three inheritance models: dominant (comparing major allele homozygotes to heterozygotes and minor allele homozygotes), recessive (comparing major allele homozygotes and heterozygotes to minor allele homozygotes), and over-dominant (comparing to major and minor allele homozygotes to heterozygotes).’*

In the statistical analysis section, it is important to mention how the normality of the distribution in its quantitative variables was tested, as well as the calculation of statistical power to detect the genetic association with DENV infection. Additionally, when considering the evaluation of multiple variations, the correction factor in the statistical analysis (such as the Bonferroni test) should be considered.

**Response:** We thank the reviewer for his constructive suggestions. The normality of quantitative variables was tested by Shapiro-Wilk test and re-confirmed by skewness and kurtosis measurements. The Benjamini-Hochberg procedure was applied for multiple testing to avoid type I error with the false discovery rate equal to 0.05.

‘*Statistical analysis: Data was analyzed and visualized using the R software version 4.3.2 (http://www.r-project.org). A p-value < 0.05 was considered statistically significant. Clinical and demographic data were presented either as median or mean values with range for quantitative variables and absolute numbers with percent for categorical variables. The normality of distribution in the quantitative variables was tested using the Shapiro–Wilk test. Categorical data were compared using Chi-square or Fisher's exact tests, while continuous variables were compared using t-test or Kruskal-Wallis test as appropriate. Dunn’s test was applied as post-hoc pairwise tests with Bonferroni adjustment. The Benjamini-Hochberg Procedure was applied for multiple testing due to the increased risk of type I error with false discovery rate equal to 0.05*.’

Also, in the tables footnote, this has been added as below

‘*Odds ratio and p-value calculated by using binary logistic regression model adjusted for age and sex. P-values were adjusted by the Benjamini-Hochberg procedure*.’

The most worrying part is the description of the results since it lacks clarity and is poorly organized. In short, the last two ideas in the first paragraph refer to the same data (duplicated). The findings found by each inheritance model (codominant 1 and 2, dominant, recessive and over dominant) are not described.

**Response:** We thank the reviewer for his constructive suggestions. The results were reanalyzed and reinterpreted to provide a more accurate and detailed account of the findings, including the adjusted p-values in the analysis of genetic associations with dengue.

The data corresponding to the polymorphic site -1082 in the control group in Table 3 is erroneous. Please correct.

**Response:** We thank the reviewer for his constructive suggestions. Data was revised and corrected.

Table 5 shows the number of haplotype observations in patients with DEN vs Controls. Could you confirm that the data is correct?

**Response:** We thank the reviewer for his constructive suggestions. Haplotype distribution was revised and corrected.

From beginning to end, the incidence of dengue in two outbreaks in 2021 and 2022 is mentioned, but the descriptive analysis never captures the stratification of the population in these two episodes. This analysis can provide information on the possible change in circulation of a serotype that may be associated with severity and conditioned by the genetic load of the host, for example.

**Response:** We thank the reviewer for his constructive suggestions. A change in the circulation of DENV serotypes was observed and elaborated in the discussion, in comparison with earlier outbreaks. The revised version has now as

*‘The main DENV serotypes circulating during the 2021-2022 outbreak were DENV-2 and DENV-1, followed by DENV-4. In northern Vietnam, the distribution of DENV-1,-2 serotypes have undergone changes in recent years. DENV-1 was previously observed as the predominant serotype during the years 2017-2019 (18). However, during the outbreaks in 2019-2020, there was a shift to DENV-2 dominance (19), a trend that persisted throughout the 2021-2022 period, as observed in our study. The DENV-1,-2,-4 serotypes were persisting in the northern population compared to other regions, where all four serotypes co-existed (20). It is well described that DENV-2 is associated with severe disease progression (2), and more than 61% of DENV-2 infections were observed in patients with DWS and severe dengue in our study. This finding is consistent with data from previous outbreaks that posed an increased burden on public health (21). While DENV-3 serotype were not observed in our study, this appears to be more regional in Vietnam, as reported in previous outbreaks (20).*’

To enrich your analysis and considering that in some populations the polymorphic site -1082 in the promoter region of the gene that codes for IL-10 is null, you could stratify your study population (cases) by genotype and verify if there are differences in the host genetics load vs. cytokine levels.

Response: We thank the reviewer for his constructive suggestions.

- The relationship between genetic variants and IL-10 levels was investigated, but no significant association was observed at either genotype or haplotype levels. However, the ACC haplotype (rs1800896 -1082A, rs1800871 -819C, rs1800872 -592C) exhibited a relatively low IL-10 levels compared to the others, as illustrated in Figure 3.

Reviewer #2: The analysis was well presented and matches the analysis plan

**Response**: Thank YOU

The results were clear and well presented

**Response**: Thank YOU

The authors should remove the dividing horizontal lines in all the tables to match the best practices of table presentation.

**Response**: The suggestion of the reviewer was considered in the revised manuscript.

The images have sufficient quality and clarity

**Response**: Thank YOU

--------------------

Reviewer #1: The conclusions do not support the findings presented in this research, do not clearly describe the **limitations of the analysis**, and do not highlight the usefulness of the data to advance the understanding of the close relationship between host genetic load and DENV genetic variability.

**Response**:

- The limitation of the manuscripts is the small sample size, as this cross-sectional study concentrated on hospitalized patients with dengue only. Conversely, this also be seen as a strength of the study, as the most important group of dengue infection (those who are symptomatic and hospitalized) was investigated.

- The study highlighted: (1) Epidemiological visions of circulating DENV in the 2021 and 2022 outbreaks. (2) Insights into the distribution of *IL-10* SNPs (rs1800896, rs1800871, rs1800872) in the Vietnamese population. (3) The first study to associate these *IL-10* SNPs with dengue in Vietnam yielded results indicating a potential protective or risk-increasing role for *IL-10* genetic variants in dengue. This finding opens the door to further studies aimed at elucidating the pathogenesis of the disease. (4) The association between IL-10 plasma levels and dengue severity was confirmed and IL-10 levels could be considered as an indicator of disease deterioration.

Reviewer #2: The conclusion supported the objectives the authors stated in the introductory section

The limitations were described by the authors as insufficient sample size and inability to investigate other relevant cytokines

**Response**: Thank YOU

The authors did not discuss the usefulness of these observation to the control of the Dengue in the study area or at large. They should also further discuss how this result of this study can be translated in Dengue control.

**Response**: Thank You. This has been added

- The association between IL-10 plasma levels and dengue severity was confirmed, and IL-10 levels may be further considered as an indicator of disease deterioration. Moreover, the findings regarding *IL-10* genetic variants associated with protection from dengue (haplotype GTA), as also observed in other studies of schistosomiasis and malaria, indicate the potential role of IL-10 genetic variants in the immune response upon infection. A more extensive investigation into population may assist in defining susceptibility to other infectious diseases, thereby providing relevant findings for the public health sector.

--------------------

<b>Editorial and Data Presentation Modifications?</b></br><br/>

Use this section for editorial suggestions as well as relatively minor modifications of existing data that would enhance clarity. If the only modifications needed are minor and/or editorial, you may wish to recommend “Minor Revision” or “Accept”.

Reviewer #1: (No Response)

Reviewer #2: (No Response)

--------------------

<b>Summary and General Comments</b></br></br>

Use this section to provide overall comments, discuss strengths/weaknesses of the study, novelty, significance, general execution and scholarship. You may also include additional comments for the author, including concerns about dual publication, research ethics, or publication ethics. If requesting major revision, please articulate the new experiments that are needed.

**Reviewer #1:**

**Response**: Thank YOU. All addressed as above

**Reviewer #2**: The authors reported the incident and serotypes of dengue virus in two outbreaks that occurred in Vietnam in 2021 and 2022. They also compared the cytokine IL-10 profile in the dengue positive patients in comparison to negative control. The study is well designed, executed and presented. The authors will need to attend to some minor observations

1. the public health impact and how the result could be translated to aid the control of dengue should be stated in the discussion. They can also include the appropriate sustainable development goals that the study addresses

**Response**: Thank YOU. We wish not to extrapolate only based on our results.

2. it will be helpful if the authors can also present a figure to show the IL-10 level based on the genotypes observed in the study

**Response**: Thank YOU. The relationship between genetic variants and IL-10 levels was investigated, but no significant association was observed at either genotype or haplotype levels. However, the ACC haplotype (rs1800896 -1082A, rs1800871 -819C, rs1800872 -592C) exhibited a relatively lower IL-10 level compared to the others, as depicted in Figure 3.

3. The horizontal lines in the table should be removed to follow the best practice of table presentation

**Response**: Thank YOU. This has been corrected

4. The sentence under "genotyping of interleukin-10 variants" should be corrected. n=299 should be after the word patients while n=300 should be after the word controls.

**Response**: Thank YOU. The suggestion of the reviewer was considered in the new manuscript.

--------------------

PLOS authors have the option to publish the peer review history of their article (<a href="https://journals.plos.org/plosntds/s/editorial-and-peer-review-process#loc-peer-review-history" target="_blank">what does this mean?</a>). If published, this will include your full peer review and any attached files.<br><br>

If you choose “no”, your identity will remain anonymous but your review may still be made public.<br><br>
