# Supplementary material for: Characterization of dengue patients in Vietnam: Clinical, virological, and IL-10 profiles during 2021– 2022 outbreaks
Source: PLoS Negl Trop Dis. 2025 Mar 28;19(3):e0012954. doi: 10.1371/journal.pntd.0012954 (PMC11977983; doi:10.1371/journal.pntd.0012954)
Supplement: Attachment — Submitted filename: Response to Reviewers.docx [file pntd.0012954.s002.docx]

PNTD-D-24-00849

Characterization of Dengue Patients in Vietnam: Clinical, Virological, and IL-10 Profiles during 2021- 2022 outbreaks

-----------------------------

**Reviewers' comments:**

**Methods**

Reviewer #1: The objective of the study is clearly stated, and the population well described. There is no ethical concern and the statical analysis was well presented. The authors did not show how they arrived at the sample size. I assumed a convenient sampling method was employed. The author should state clearly the sampling technique that was used.

**Response**: We thank the reviewer for the constructive comment. Our study aimed to capture cases of dengue during two consecutive seasonal outbreaks in a defined geographic area (Hanoi, Vietnam). This method allowed us to include a representative sample of patients admitted to the central hospital during these outbreaks, providing valuable insights into the genetic associations within the context of seasonal variations in dengue incidence. We have now explicitly stated this sampling technique in the Methods section, as follows:

- “*The study employed a convenience sampling method, including patients who were admitted to the central hospital in Hanoi, Vietnam, during two consecutive seasonal dengue outbreaks between October 2021 and December 2022.*”.

Reviewer #2: This paper describes an important topic that is growing in impact as numbers of dengue cases are rising in many disparate geographic areas of the globe. Its strengths include the numbers of cases studied, the inclusion of clinically severe cases and the detailed analysis of Il-10 promotor polymorphism associations with disease severity allowing for dengue serotypes. An additional strength is the exclusion of infection by closely related flaviviruses.

**Response**: We sincerely appreciate your positive feedback and recognition of the strengths of our study. Your acknowledgment of the importance of the topic, the robust study design, and the detailed analysis of IL-10 promoter polymorphism associations with dengue severity are very encouraging. We are especially grateful for your note on the inclusion of clinically severe cases, the consideration of dengue serotypes, and the exclusion of infections by related flaviviruses, which we believe are critical to the study's validity and relevance.

**Results**

Reviewer #1: The analysis plan is good and well presented. The figures and tables are sufficient. The author might consider removing figure 1b because the same data is well presented in Table 1.

**Response**: We thank the reviewer for this suggestion. Figure 1b was removed accordingly.

Reviewer #2: The analysis of associations for polymorphisms using logistic regression models and different assignments of gene transmission (*dominant, recessive and so on) is appropriate, and the findings are of interest. The results are clearly presented.

**Response**: Thank you for your thoughtful evaluation of our manuscript and for acknowledging the appropriateness of our analysis using logistic regression models with varied gene transmission assignments (dominant, recessive, etc.). We are glad that you found the findings of interest and that the clarity of the results presentation met your expectations.

**Conclusions**

Reviewer #1: The conclusion was clear, and the study was well discussed but the public health implication is not clearly stated

**Response**: We thank the reviewer for the constructive comment. This has been now addressed in the revised version as below:

-“ *In summary, this study underscores the importance of understanding genetic predispositions and immune response mechanisms in dengue severity, particularly in endemic regions like Vietnam. The identification of IL-10 plasma levels and genetic variants, specifically the protective role of the -1082G allele, provides valuable insights into host-pathogen interactions and susceptibility to severe dengue. Such findings can guide the development of targeted public health strategies, including early risk stratification, personalized treatment approaches, and the design of immunogenetic-based interventions. Furthermore, the consistent dominance of DENV-2 and the associated higher severity highlights the need for serotype-specific monitoring and vaccination campaigns to mitigate the growing burden of dengue in Southeast Asia. This integrative approach, combining clinical, genetic, and serotype data, is pivotal in enhancing preparedness and response efforts in dengue-endemic regions.*”

Reviewer #2: As mentioned there may be other chemo-cytokines that are relevant to this topic and further comments on this topic may be helpful for context, including in the discussion. Are there any in vitro data looking at Il10 promotor polymorphisms in ex vivo experiments that might shed light on the functional relevance of these polymorphisms? For example, on the timing of release of Il-10 (PMCID: PMC11508614 DOI: 10.3390/ijms252011238) and with relevance to another earlier model of infection, which the authors might consider worth mentioning in discussion (reference abstract appended). Lancet. 1997 Jan 18;349(9046):170-3. doi: 10.1016/s0140-6736(96)06413-6. Genetic influence on cytokine production and fatal meningococcal disease R G Westendorp 1 , J A Langermans, T W Huizinga, A H Elouali, C L Verweij, D I Boomsma, J P VandenbrouckeAffiliations expand PMID: 9111542 DOI: 10.1016/s0140-6736(96)06413-6

Erratum inLancet 1997 Mar 1;349(9052):656. Vandenbrouke JP [corrected to Vandenbroucke JP]

Abstract

Background: To assess the genetic influence on cytokine production and its contribution to fatal outcome, we determined the capacity to produce tumour necrosis factor-alpha (TNF alpha) and interleukin-10 (IL-10) in families of patients who had had meningococcal disease.

Methods: We studied 190 first-degree relatives of 61 patients with meningococcal disease; we also studied 26 monozygotic twins. Production of cytokines was determined during endotoxin stimulation of whole-blood samples ex-vivo. Heritability was estimated in a pedigree-based maximum-likelihood model. DNA was typed for the G to A transition polymorphisms at position -308 and -238 in the TNF gene promoter.

Findings: Heritability in monozygotic twins was 0.60 for the production of TNF and 0.75 for the production of IL-10. Families with low TNF production had a tenfold increased risk for fatal outcome (OR 8.9, 95% CI 1.8-45), whereas high IL-10 production increased the risk 20-fold (19.5, 2.3-165). Families with both characteristics had the greatest risk. The transition polymorphisms in the TNF gene promoter were not associated with outcome.

Interpretation: Genetic factors substantially influence production of cytokines. An innate anti-inflammatory cytokine profile may contribute to fatal meningococcal disease.

**Response**: We thank the reviewer for the constructive comment and valuable suggestions. All suggested references were taken into account to discussion on the current findings and the discussed as below with provided references:

-“*Upon monocyte infection with DENV, the expression of IL-10 gradually rises over time and more in the case of ADE (22). Secondary infections trigger an earlier and higher release of IL-6 and IL-10 compared to primary infections, indicating that pre-existing immune memory accelerates cytokine production in subsequent infections (25). Several transcription factors are involved in the modulation of IL-10 transcription, and approximately 75 % of the variation in IL-10 secretion capacity derives from genetic factors (26, 27). Westendorp et al. further demonstrated a strong genetic influence on cytokine production, including IL-10, upon ex vivo stimulation (28). This suggests that genetic factors may influence IL-10 levels, thereby affecting the severity and susceptibility to dengue*.”

___________________________

Editorial and Data Presentation Modifications?

Reviewer #1: (No Response)

Reviewer #2: Minor Revision

----------------------------------------

Summary and General Comments

Use this section to provide overall comments, discuss strengths/weaknesses of the study, novelty, significance, general execution and scholarship. You may also include additional comments for the author, including concerns about dual publication, research ethics, or publication ethics. If requesting major revision, please articulate the new experiments that are needed.

Reviewer #1: Overall the study is well executed and the paper well written

The inheritance model analysis section should be part of the statistical analysis.

**Response**: Thank you for your positive feedback and constructive suggestion. We agree that the inheritance model analysis should be more clearly integrated into the statistical analysis section. We have revised the manuscript accordingly to ensure that the inheritance model is discussed within the context of the statistical methods, providing a more cohesive flow for readers, as below:

*‘Allele, genotype, or haplotype frequencies were determined by simple gene counting and deviations from Hardy-Weinberg equilibrium were tested. The association between IL-10 genetic variants and dengue was assessed using logistic regression, adjusting for age and sex, under three inheritance models: dominant (comparing homozygotes for the major allele to heterozygotes and homozygotes for the minor allele), recessive (comparing homozygotes for the major allele and heterozygotes to homozygotes for the minor allele), and over-dominant (comparing heterozygotes to homozygotes for both the major and minor alleles). The IL-10 haplotypes were estimated using the R package "SNPassoc" (16) version 2.1.0 and "haplo.stats" (17) version 1.9.3. The linkage disequilibrium (LD) analysis was performed with the program Haploview v.4.1’*

Reviewer #2: The paper is well written.

**Response**: Thank You.

PLOS authors have the option to publish the peer review history of their article (what does this mean?). If published, this will include your full peer review and any attached files.

If you choose “no”, your identity will remain anonymous but your review may still be made public.

Do you want your identity to be public for this peer review? For information about this choice, including consent withdrawal, please see our Privacy Policy.

Reviewer #1: No

Reviewer #2: No
